# Attention and Suggestions on the Study of Symptom Clusters in ICI Treatment

**DOI:** 10.1002/hsr2.72714

**Published:** 2026-06-19

**Authors:** Kong Qingfei, Xiao Wen

**Affiliations:** ^1^ Capital Medical University Affiliated Huairou Teaching Hospital, Beijing Huairou Hospital Beijing China

## Transparency Statement

The lead and Corresponding author (Kongqingfei) affirms that this manuscript is an honest, accurate, and transparent account of the study being reported; that no important aspects of the study have been omitted; and that any discrepancies from the study as planned have been explained.

To Editor: My colleagues and I have recently read and deeply discussed the research article titled “Patient‐Reported Symptom and Symptom Clusters During Immune Checkpoint Inhibitor Therapy for Cancer Patients: A Cross‐Sectional Survey” published in your journal. We are writing to express our deep concern and high appreciation for this study. Firstly, we sincerely agree with the entry point of the research [1]. The study keenly captures a critically important yet underexplored clinical issue in immune checkpoint inhibitor (ICI) therapy—the patient‐reported symptom burden and its clustering phenomenon. The research clearly points out that traditional clinician‐reported adverse event assessment tools may overlook the subjective experiences of patients, while Patient‐Reported Outcomes (PRO) can provide a more sensitive and comprehensive assessment [2, 3]. This perspective places patients at the center of cancer care and holds significant value for optimizing patient‐centered supportive treatment strategies. Furthermore, the study focuses on the concept of “symptom clusters,” going beyond the management of isolated symptoms to explore the underlying common biological mechanisms behind multiple co‐occurring symptoms, providing a novel theoretical framework for the future development of mechanism‐driven and more effective interventions. Secondly, we commend and pay attention to the experimental research methods employed by the research team. The study is designed as a cross‐sectional observational survey conducted at Guang'anmen Hospital in China, including 213 cancer patients receiving ICI therapy, providing valuable data for understanding ICI‐related symptoms in real‐world clinical settings. The highlights of the research methodology are particularly prominent: Standardized application of PRO tools: The study used validated PRO assessment tools to systematically collect data on symptom severity, functional interference, and health‐related quality of life, ensuring the objectivity and scientific nature of the assessment. Rigorous symptom cluster analysis methods: The study employed exploratory factor analysis to identify symptom clusters and set strict inclusion criteria (e.g., moderate to severe symptom incidence > 15%, factor loadings > 0.5), while conducting KMO tests and Bartlett's test of sphericity to verify data appropriateness, reflecting the rigor of statistical analysis. Multidimensional correlation analysis: The study not only performed descriptive statistics but also analyzed factors influencing symptom burden (such as ECOG performance status and early‐stage immunotherapy) through multiple linear regression, and examined the relationship between symptom burden and quality of life through Pearson correlation analysis, adding depth to the conclusions. Based on our in‐depth consideration of this study, we would like to propose the following suggestions for the authors and future related research:Advance longitudinal research design: As a cross‐sectional survey, this study successfully depicts the symptom profile at a single time point. We suggest that future research establish dynamic assessments at multiple time points to track the evolution of symptoms and symptom clusters over the course of treatment. This will help clarify the occurrence and development patterns of symptom clusters and their potential causal relationships with treatment responses and long‐term prognosis.

Deepen mechanism exploration and biomarker integration: The study's conclusions indicate that different symptom clusters may have different biological mechanisms. We strongly agree with this viewpoint and suggest that subsequent research could attempt to integrate biomarker detection (such as inflammatory cytokine profiles, autoantibodies, etc.) to empirically explore the shared pathophysiological basis behind symptom clusters, thereby providing direct evidence for the development of targeted interventions [4].

Expand research population and refine treatment pattern analysis: In this study, lung cancer patients accounted for a high proportion (75.1%). Future research could expand the sample size to include patients with various cancer types to validate the universality of symptom cluster patterns [5]. Additionally, further differentiation and analysis of symptom characteristics between patients receiving ICI monotherapy and combination therapy (such as combined chemotherapy) could help clarify whether symptoms arise from ICI itself or the influence of the combination treatment regimen.

In conclusion, we believe this study is a high‐quality and forward‐looking work that successfully applies PRO and symptom cluster theory to the field of ICI therapy, pointing out important directions for clinical practice and subsequent research. We look forward to seeing the team achieve more groundbreaking results on this solid foundation, and we thank your journal for continuously publishing excellent research that promotes advancements in clinical oncology supportive care.

## Author Contributions

**Xiao Wen:** validation, investigation, data curation, writing – original draft, visualization. **Kong Qingfei:** conceptualization, methodology, formal analysis, writing – original draft, software, data curation, supervision, writing – review and editing.

## Funding

The authors have nothing to report.

## Conflicts of Interest

The authors have no conflicts of interest.

## Data Availability

Data sharing not applicable to this article as no datasets were generated or analysed during the current study.

## References

[1] S. Ren , J. Yang , H. Xu , et al., “Patient‐Reported Symptom and Symptom Clusters During Immune Checkpoint Inhibitor Therapy for Cancer Patients: A Cross‐Sectional Survey,” Health Science Reports 9 (2026): 1–9, 10.1002/hsr2.71708. PMC12802557 41542332

[2] C. L. Bi , D. B. Kurland , R. Ber , et al., “Digital Biomarkers and the Evolution of Spine Care Outcomes Measures: Smartphones and Wearables,” Neurosurgery 93, no. 4 (2023): 745–754, 10.1227/neu.0000000000002519. 37246874

[3] S. Y. Ng , K. L. Colborn , L. Cambridge , et al., “Induction Chemotherapy Reduces Patient‐Reported Toxicities During Neoadjuvant Chemoradiation With Intensity Modulated Radiotherapy for Rectal Cancer,” Clinical Colorectal Cancer 18, no. 3 (2019): 167–174, 10.1016/j.clcc.2019.04.001. 31104990 PMC6817304

[4] Y. Wang , L. Fang , K. Zhou , et al., “Symptom Burden and Clusters During Chemotherapy in Patients With Lung Cancer,” World Journal of Surgical Oncology 22, no. 1 (2024): 309, 10.1186/s12957-024-03594-0. 39578897 PMC11583389

[5] Y. Guzman‐Prado , J. Ben Shimol , and O. Samson , “Body Mass Index and Immune‐Related Adverse Events in Patients on Immune Checkpoint Inhibitor Therapies: A Systematic Review and Meta‐Analysis,” Cancer Immunology, Immunotherapy 70, no. 1 (2021): 89–100, 10.1007/s00262-020-02663-z. 32648164 PMC10991299

